# Effects of Erector Spinae Plane Block on Postoperative Pain and Quality of Recovery Questionnaire Scores in Video-Assisted Thoracoscopic Surgery: A Randomized Controlled Study

**DOI:** 10.7759/cureus.36089

**Published:** 2023-03-13

**Authors:** Meliha Orhon Ergun, Ecem Guclu Ozturk, Seniyye Ulgen Zengin

**Affiliations:** 1 Anesthesiology and Reanimation Department, Marmara University Medical School, Istanbul, TUR; 2 Anesthesiology and Reanimation Department, Marmara University Pendik Education and Research Hospital, Istanbul, TUR; 3 Anesthesiology and Reanimation Department, Marmara University School of Medicine, Istanbul, TUR

**Keywords:** opioid, postoperative pain management, opioid-free anesthesia, erector spinae plane block (espb), lobectomy, video-assisted thoracic surgery (vats)

## Abstract

Objectives: Opioid-free anesthesia with erector spinae plane block (ESPB) has the potential to decrease perioperative opioid need, thereby potentially reducing related complications. This study aimed to compare opioid-free anesthesia with ESPB and standard opioid-based balanced anesthesia in patients undergoing video-assisted thoracic surgery (VATS) in terms of postoperative opioid need (through patient control analgesia) as well as postoperative pain management, recovery quality, and opioid-related side effects.

Methods: Seventy-four patients, ranging in age from 18 to 75 years, who underwent lobectomy with VATS were included in this randomized-controlled study. The opioid-free group had ESPB, and no opioid was used during anesthesia maintenance. The opioid group received standard anesthesia with opioid use. Postoperative morphine requirement, postoperative pain as measured by the visual analog scale (VAS), intraoperative vital parameters, recovery quality using the Quality of Recovery-40 (QoR-40) questionnaire, and opioid-related complications were compared between groups.

Results: The opioid-free group received a significantly lower total dose of morphine during the first 24 postoperative hours through patient-controlled analgesia (PCA) when compared to the opioid group (7.3±3.4 vs. 21.7±7.9 mg, p<0.001). In addition, the opioid-free group had significantly better postoperative pain scores and QoR-40 scores (184.3±7.5 versus 171.2±6.4, p<0.001), shorter times to mobilization (5.5±0.8 versus 8.1±1.1 hours, p<0.001), and oral intake (5.8±0.6 versus 6.4±0.6 hours, p<0.001), as well as less frequent opioid-related side effects.

Conclusion: The findings of this study suggest that opioid-free anesthesia with ESPB represents a promising option for patients undergoing lobectomy with VATS. It has the potential to decrease postoperative opioid need, improve postoperative pain management, and reduce opioid-related unwanted consequences.

## Introduction

Neuropathic pain following video-assisted thoracic surgery (VATS) is still an important postoperative problem, although it is less marked when compared to the thoracotomy approach [1]. Pain management following VATS is particularly important for the prevention of respiratory complications. Opioid-based anesthesia is widely used in VATS for perioperative analgesia; however, high doses of intraoperative and postoperative opioid administration have the potential to negatively affect postoperative recovery since opioids have been associated with respiratory depression, hyperalgesia, nausea, and vomiting in surgical patients [2-5]. On the other hand, opioid-free anesthesia has been shown to decrease postoperative analgesia and/or morphine requirement, thereby potentially reducing opioid-related complications and side effects [6-8].

Ultrasound-guided erector spinae plane block (ESPB) is a recently defined regional anesthesia technique where local anesthetic is injected into the erector spinae muscle and fascial plane and the agent is allowed to diffuse in caudal and cranial directions. This technique has been successfully used in thoracic surgery and trauma, breast surgery, abdominal surgery, and extremity surgery for the purpose of intraoperative regional anesthesia as well as postoperative analgesia [9]. Regional block methods, including ESPB, have been mostly used for postoperative analgesia after VATS, and they have been shown to provide effective postoperative analgesia and reduce morphine consumption [10-18]. However, the evidence for the use of ESPB or other block techniques for the purpose of anesthesia during the VATS procedure is relatively scarce [19-20]. Opioid-free anesthesia with the aid of ESPB may help reduce the total and postoperative opioid load in patients undergoing VATS, thereby favorably contributing to postoperative recovery.

This study aimed to compare opioid-free anesthesia with the aid of ultrasound-guided ESPB with standard anesthesia with opioid use in patients undergoing lobectomy with VATS in terms of postoperative opioid need and pain management, recovery quality, as well as opioid-related complications.

## Materials and methods

Patients

Forty-eight male and 29 female patients who are 42-70 years old with ASA (American Society of Anesthesiologists) physical status ≤3 who underwent lobectomy with VATS and provided informed consent for study entry were included in this randomized-controlled study. Patients with contraindications to either of the anesthesia methods, chronic pain, allergy to local anesthetics, spinal deformity, mental or psychiatric problems preventing cooperation, as well as patients using opioids or anticoagulants were excluded from the study. Randomization was carried out at a ratio of 1:1 using the closed-envelope method. Random allocation cards were made using computer-generated random numbers and placed in sealed envelopes (opaque and non-resealable). Before the procedure, a sealed envelope was opened by the anesthesiologist in the operating room, and the procedures were performed according to the group specified in the paper, either an opioid-free anesthesia group or an opioid-based anesthesia group. In addition, the following patients were not included in the analyses: patients in whom ESPB was unsuccessful; patients who required opioids during the procedure (for the opioid-free group); and patients requiring a switch to open surgery (for both groups). This study was approved by the local ethics committee, and all patients gave written informed consent prior to study entry. The study was conducted in accordance with the ethical standards of the institutional ethics committee and with the 1964 Helsinki Declaration and its later amendments or comparable ethical standards. Ethical approval: This study was approved by the ethics committee of the Marmara University Faculty of Medicine (number 09.2022.100; date: 07.01.2022). Trial registration: NCT05321576 (ClinicalTrials.gov).

Anesthesia management

In addition to standard anesthesia monitoring (electrocardiography, non-invasive blood pressure, and oxygen saturation measurements), all patients were monitored with the bispectral index (BIS) and the Analgesia Nociception Index (ANI). ANI and BIS were monitored to objectively evaluate perioperative pain and anesthesia depth, respectively. Anesthesia management was provided with the same volatile agent, sevoflurane, in both groups, with added intravenous remifentanil infusion in the "opioid group," and intravenous dexmedetomidine infusion in the "opioid-free group." The infusion rate of remifentanil and dexmedetomidine was titrated to keep ANI monitoring values between 50 and 70. The BIS value was targeted in the range of 40 to 60. Table 1 summarizes the anesthesia management of the two groups.

**Table 1:** Summary of intraoperative anesthesia and postoperative pain management of the two groups BIS: bispectral index, ANI: Analgesia Nociception Index, ESPB: erector spinae plane block, PCA: patient controlled analgesia

	Opioid group	Opioid-free group
Block	None	ESPB
Anesthesia induction	Propofol, remifentanil, rocuronium	Propofol, ketamine, rocuronium
Anesthesia maintenance	Remifentanil	Dexmedetomidine
Intraoperative monitoring	BIS, ANI	BIS, ANI
Postoperative analgesia	PCA	PCA

Opioid-free anesthesia group (with ESPB)

Before induction of anesthesia, ESPB was carried out on one side (at the side of the operation) by an experienced anesthesiologist in a sitting position and under ultrasound guidance. The injection site was identified at 3 cm lateral to the T5 spinous process, and injection was done using the in-plane technique. The needle was advanced in a cranio-caudal direction, and 1-2 ml of saline was injected to separate the erector spinae muscle from the transverse process. Following the separation, 20 ml of 0.5% bupivacaine and 100 mg (5 ml) of lidocaine were injected. No analgesic or sedative was used during the procedure. Anesthesia was induced with propofol (2 mg/kg), ketamine (2 mg/kg), and rocuronium (0.6 mg/kg). This group did not take any opioids during the operation but instead received continuous dexmedetomidine infusion at an initial dose of 0.4 µg/kg/h, and the dose was adjusted to keep the BIS value between 40 and 60 and the ANI between 50 and 70.

Opioid anesthesia group

Anesthesia was induced with propofol (2 mg/kg), remifentanil (1 µg/kg), and rocuronium (0.6 mg/kg). This group received continuous remifentanil infusion with an initial dose of 0.5 µg/kg/h, and the dose was adjusted to keep BIS <50 and ANI >50 throughout the operation. After the surgical procedure was completed before awakening, both groups received intravenous 1 g of paracetamol.

Assessments

Groups were compared in terms of study outcomes. The primary outcome was postoperative morphine requirement (during the first and second 24 hours). Secondary outcomes were as follows: postoperative pain (as measured by the visual analog scale [VAS]), need for rescue analgesia, anesthesia-related complications, indicators for postoperative recovery (Quality of Recovery-40 [QoR-40] questionnaire assessment, time to mobilization [hours], time to oral intake [hours], and duration of hospitalization [days]), and changes in intraoperative measurements including heart rate (HR), mean arterial blood pressure (MAP), oxygen saturation (SpO_2_), BIS, and ANI.

The QoR-40 is a self-rated 40-item questionnaire evaluating the quality of postoperative recovery and health status early after surgery [21]. Its version validated for the Turkish language was used [22]. The questionnaire was administered to each patient during their postoperative hospital stay before discharge. Higher points indicate better postoperative recovery and health status.

Postoperative pain management

Patients received intravenous patient-controlled analgesia (PCA), where 4 mg of morphine was self-delivered by the patient with each application with a lockout time of 15 minutes. When necessary, rescue analgesia (tramadol or paracetamol) was provided. Morphine doses and rescue analgesia needs were recorded. Immediately after awakening and at 6, 12, 24, and 48 postoperative hours, the pain was evaluated using a 10-point VAS.

Statistical analysis

Sample size estimation revealed that a total of 33 patients per group would be necessary to detect a mean difference of at least 2.5 mg in morphine equivalent consumption within the first 24 hours between the two treatment groups, with an alpha error of 0.05 and a beta value of 0.1 (power = 0.9). Considering the 10% dropout rate, the inclusion of 37 patients in each group was planned. For data analysis, Statistical Package for Social Sciences (SPSS) version 21 software (IBM Corp., Armonk, NY) was used. The normality of continuous variables was tested using graphical methods and hypothesis tests. Between-group comparisons of continuous variables were done using the student t-test for independent samples or the Mann-Whitney U test, depending on the data distribution. The Pearson chi-square test or Fisher’s exact test was used for between-group comparison of categorical variables. A two-way analysis of variance (ANOVA) test for repeated measurements was used to examine the differences between groups in terms of change in intraoperative variables and VAS scores over time. The comparison of groups at different time points was done with a Mann-Whitney U test or a student t-test, where appropriate. Two-sided p-values <0.05 were considered indications of statistical significance.

## Results

Patients

Out of 77 patients enrolled in this study, the ESP block failed in one patient, and two patients could not give a proper anamnesis after surgery. These patients were excluded from follow-up. Seventy-four patients were included in the analysis. Demographic characteristics and clinical data are shown in Table 2. Patients in the "opioid-free group" had a significantly higher body mass index (BMI) than the opioid group (p=0.011). Sex distribution and age were similar between the groups.

**Table 2:** Demographic and clinical data of the groups. M: male; F: female; BMI: body mass index; QoR-40: Quality of Recovery-40 questionnaire. Unless otherwise stated, data presented as mean ± standard deviation.

	All patients	Opioid group	Opioid-free group	P-value
	(n=74)	(n=37)	(n=37)	
Demographics				
Age, years	56.4±14.0	56.6±16.7	56.2±10.9	0.222
Sex, M/F	46/28	25.Ara	21/16	0.338
BMI, kg/m^2^	26.5±4.7	25.0±4.3	28.0±4.6	0.004
Recovery parameters				
QoR-40 score	177.7±9.6	171.2±6.4	184.3±7.5	<0.001
Time to mobilization (h)	6.8±1.6	8.1±1.1	5.5±0.8	<0.001
Time to oral intake (h)	6.1±0.7	6.4±0.6	5.8±0.6	<0.001
Duration of hospitalization (d)	4.5±1.7	4.9±2.1	4.2±1.0	0.317
Postoperative morphine consumption (mg)				
First postoperative 24 hours (0-24)	15.0±9.9	21.7±7.9	7.3±3.4	<0.001
Second postoperative 24 hours (24-48)	15.7±8.8	18.7±10.9	12.8±4.5	0.103

Intraoperative assessments

**Figure 1:**
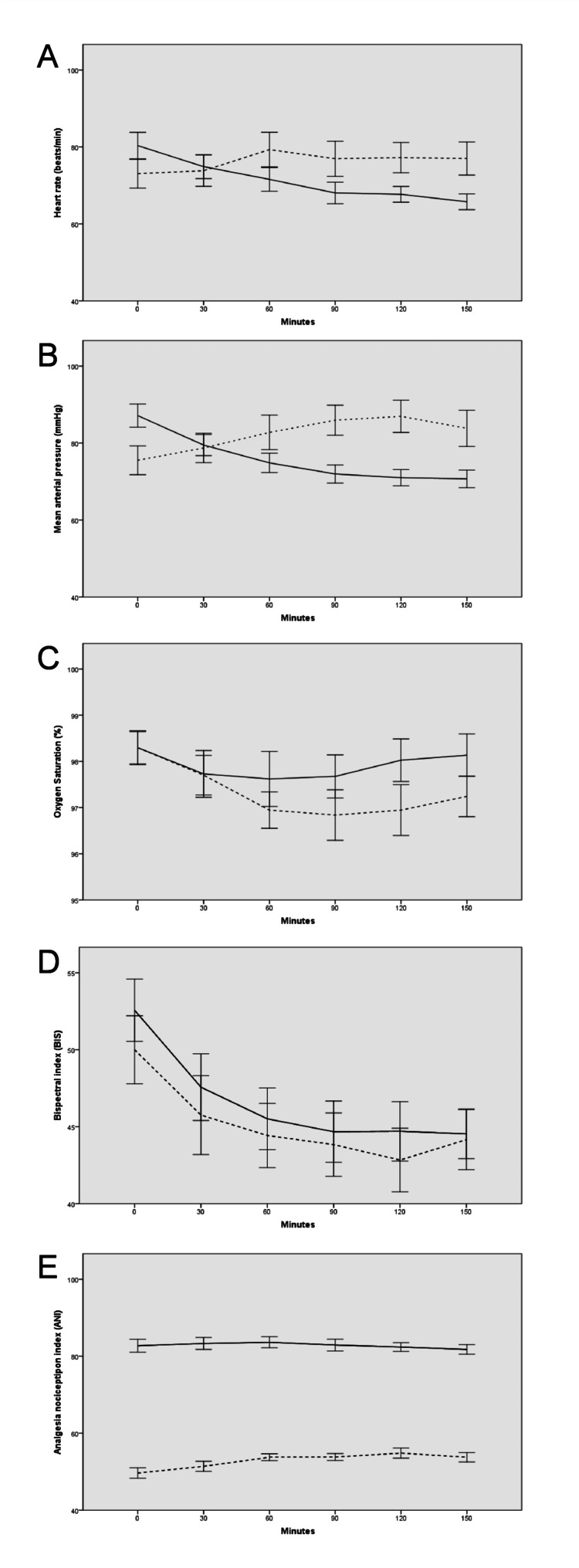
Changes in mean heart rate (A), arterial pressure (B), oxygen saturation (C), bispectral index (D), and Analgesia Nociception Index (E) over time during the operation. Error bars indicate 95% confidence intervals. Straight lines and dotted lines indicate opioid-free group and standard anesthesia group (with opioid), respectively.

The mean opioid (remifentanil) amount administered during the operation was 1.9±0.4 mg (median, 1.9; range, 1.3-2.8 mg) in the opioid group. Figure 1 shows intraoperative changes in heart rate, mean arterial pressure, oxygen saturation, BIS, and ANI. The two groups differed in terms of changes in HR (p<0.001), MAP (p<0.001), SpO_2_ (p=0.025), and ANI (p=0.001) over time, but not in terms of BIS changes (p=0.444).

The opioid-free group had significantly higher HR and MAP at baseline; however, at and after 60 minutes, these two parameters were significantly lower in the opioid-free group (p<0.001 for all). Oxygen saturation was lower in the opioid group at and after 60 minutes (p<0.05 for all). ANI scores were significantly higher in the opioid group when compared to the non-opioid group at all time points (p<0.001 for all).

Postoperative assessments

Changes in VAS Scores

**Figure 2:**
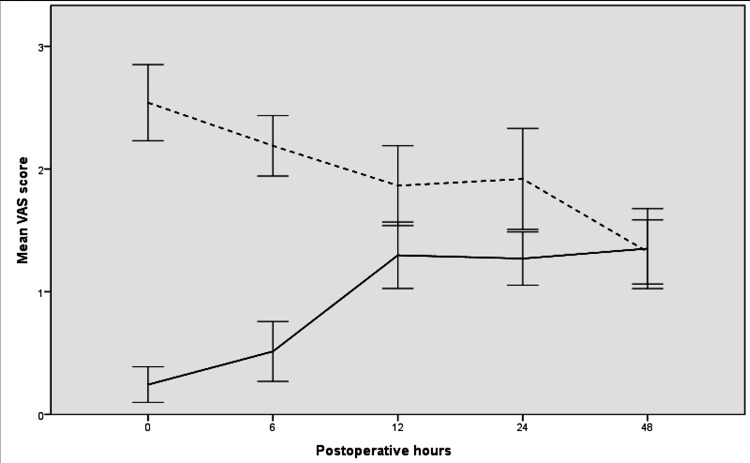
Changes in mean postoperative visual analog scale scores over time. Error bars indicate 95% confidence intervals. Straight lines and dotted lines indicate opioid-free group and standard anesthesia group (with opioid), respectively.

Figure 2 shows postoperative changes in VAS scores. The two groups differed in terms of changes in VAS scores (p<0.001). The opioid-free group had significantly lower VAS scores at all time points (p<0.05 for all), except for 48 hours (p=0.986).

Postoperative Opioid Requirement

Postoperative intravenous opioid doses administered via patient-controlled analgesia are shown in Table 2. The opioid-free group received a significantly lower total dose of morphine during the first 24 postoperative hours when compared to the opioid group (7.3±3.4 vs. 21.7±7.9 mg, p<0.001). However, the two groups did not differ regarding the morphine requirement during the next 24 hours (p=0.103). Postoperatively, intravenous tramadol 1.5 mg/kg was set as rescue analgesia. None of the patients in the opioid-free group required rescue analgesics, whereas this was required by all patients in the opioid group.

Postoperative Recovery

The opioid-free group had significantly better QoR-40 scores (184.3±7.5 versus 171.2±6.4, p<0.001), and shorter times to mobilization (5.5±0.8 versus 8.1±1.1 hours, p<0.001) and oral intake (5.8±0.6 versus 6.4±0.6 hours, p<0.001) (Table 2). However, the two groups did not differ in terms of the duration of hospitalization.

Opioid Related Side Effects

Table 3 shows the distribution of opioid-related side effects across groups. The frequencies of each side effect as well as the presence of any side effect were significantly lower in the opioid-free group (p<0.05 for all).

**Table 3:** Distribution of opioid-related side effects across groups.

Side effect	All patients (n=74)	Opioid group (n=37)	Opioid-free group (n=37)	P-value
Dyspnea	18 (24.3%)	18 (48.6%)	0 (0.0%)	<0.001
Urinary retention	17 (23.0%)	17 (45.9%)	0 (0.0%)	<0.001
Sleepiness	7 (9.5%)	7 (18.9%)	0 (0.0%)	0.011
Pruritis	13 (17.6%)	13 (35.1%)	0 (0.0%)	<0.001
Nausea	19 (25.7%)	15 (40.5%)	4 (10.8%)	0.003
Vomiting	15 (20.3%)	12 (32.4%)	3 (8.1%)	0.009
Any opioid-related side effect	36 (48.6%)	32 (86.5%)	4 (10.8%)	<0.001

## Discussion

This study examined the potential use of ESPB as a part of an opioid-free anesthesia protocol in comparison with a standard anesthesia protocol with the use of opioids in patients undergoing lobectomy with VATS. Promising findings have been obtained in terms of improved postoperative pain management with fewer opioids and better recovery, as well as fewer opioid-related side effects. To the best of our knowledge, this study is the first to use ESPB as a part of opioid-free anesthesia in VATS.

So far, several studies have examined the use of opioid-free anesthesia with or without regional blockade in VATS. A recent randomized controlled study compared opioid-free anesthesia with thoracic paravertebral block and dexmedetomidine versus opioid anesthesia in lung cancer patients undergoing VATS [23].​​​​​​ That study focused on the intraoperative effects of opioid-free anesthesia; and found similar effects in terms of intraoperative pain threshold but deeper sedation, higher blood glucose levels, and longer recovery and extubation times when compared to opioid anesthesia. In addition, dizziness and postoperative nausea/vomiting were more common in the opioid anesthesia group, but the duration of hospitalization was similar. The study by Selim et al. compared opioid-free anesthesia with dexmedetomidine, lidocaine, and ketamine with opioid anesthesia with remifentanil and morphine in terms of postoperative pain management in patients undergoing VATS [24]. Opioid-free anesthesia was associated with lower cumulative morphine consumption during the postoperative 48 hours and better postoperative pain control.

The use of ESPB or other regional blockade techniques for the purpose of anesthesia is rare. A 20-year-old case underwent non-intubated VATS for pulmonary bullae resection under ESPB, where anesthesia maintenance was provided by propofol and remifentanil, with a good intraoperative course and good postoperative pain control [19]. Another study reported a serratus plane block for opioid-free VATS sympathectomy in seven patients with promising results [20].

To date, several studies have examined the use of ESPB, whether alone or in combination/comparison with other regional blockade techniques, for postoperative analgesia after VATS. Studies consistently found beneficial effects of regional blockades in terms of improved postoperative pain control and reduced opioid consumption. This is in line with the findings of the present study. Ciftci et al. found that a preoperative single-shot ESPB was associated with reduced opioid consumption and improved VAS scores at all time points [12]. Similarly, Shim et al. found better pain control within the six hours after VATS as well as a lower post-anesthesia care unit stay time, less postoperative pethidine consumption, and a lower agitation score in association with ESPB when compared to controls [25]. In the study by Piskin et al., continuous ESPB was used for the purpose of postoperative analgesia, and that modality significantly reduced opioid consumption and opioid-related side effects when compared to controls [18].

Relative analgesic efficacies of different regional blockade types, including ESPB following VATS, are somewhat controversial. Fu et al. obtained superior postoperative analgesia with paravertebral block plus ESPB when compared to ESPB alone [10]. Similarly, Zengin et al. obtained effective postoperative pain management with an acceptable amount of morphine consumption with the combination of ESPB and thoracic paravertebral block [14]. In the study by Turhan et al., three modalities were compared: ESPB, thoracic paravertebral block, and intercostal nerve block following VATS [11]. Although all modalities provided sufficient postoperative analgesia, the thoracic paravertebral block was associated with better pain control and less morphine consumption. In the study by Zhao et al., ESPB provided equivalent analgesia and quality of recovery after VATS compared to paravertebral block [16]. Takeda et al., on the other hand, found that the analgesic effect of ESPB after VATS was non-inferior to that of thoracic paravertebral block 24 hours postoperatively [13].

A recent meta-analysis with 21 randomized control studies compared regional blockade methods (thoracic paravertebral block, erector spinae plane block, serratus plane block, and intercostal nerve block) with controls in patients undergoing VATS in terms of postoperative pain control [17]. Although overall thoracic paravertebral block showed the greatest effect on opioid consumption when compared to controls, ESPB had the greatest effect on pain scores within the early period (up to six hours) when compared to controls.

To the best of our knowledge, to date, only one study has examined the effect of ESPB on postoperative recovery in patients undergoing VATS using the same evaluation tool as the present study (QoR-40) [15]. In that study, ESPB resulted in better recovery scores as well as shorter post-anesthesia care unit discharge times, less acute postoperative pain, and lower cumulative opioid consumption when compared to controls. In addition, ESPB was associated with higher patient satisfaction. The findings of that study are parallel to the findings of the present study.

Opioid-free anesthesia has the potential to reduce the total opioid load not only by avoiding opioids during the operation but also by reducing postoperative opioid needs. Opioids are a special concern due to their untoward effects, including respiratory side effects, particularly for VATS. ESPB is a relatively simple and safe technique, particularly when performed under ultrasound guidance [26]; thus, it can be a part of an opioid-free anesthesia protocol since it has been used before for the purpose of postoperative pain management as well as intraoperative regional anesthesia [9]. As a regional block, it has several advantages. It can block both somatic and visceral nerves, and the procedure is performed away from important anatomical structures such as major vessels and the pleura. It uses the transverse process as a barrier, thus avoiding needle injury to the pleura [27]. Paravertebral blocks, on the other hand, are associated with a significant risk of pleural puncture and severe pneumothorax, although the risk is lower when performed under ultrasound guidance [28].

The study has some limitations. The VAS score is significantly higher at the first 24-hour follow-up in the opioid group compared to the opioid-free group. It cannot be discriminated whether this is due to opioid-induced hyperalgesia or because of the high analgesic effect of the ESPB applied in the opioid-free group. Our postoperative follow-up lasted up to 48 hours, and we did not investigate chronic pain. However, this study was designed to compare the effectiveness of these blocks for acute pain control. Finally, the study is single-centered, and the sample size is relatively small. These are also limitations of the study.

## Conclusions

The findings of this study suggest that ESPB, when performed as a part of an opioid-free anesthesia protocol in patients undergoing lobectomy with VATS, reduces opioid consumption, improves recovery, reduces opioid-related side effects, and provides better postoperative pain management. Further studies are warranted to better clarify its role as a part of opioid-free anesthesia in VATS.
